# Cost and Utilization Implications of a Health Plan's Home-Based Palliative Care Program

**DOI:** 10.1089/jpm.2023.0401

**Published:** 2024-04-02

**Authors:** Kimberly A. Bower, Jenelle Hallock, Xiaoli Li, Tyler Kent, Liane Wardlow

**Affiliations:** ^1^ Pediatric Palliative Care, Rady Children's Hospital San Diego, San Diego, California, USA.; ^2^ Blue Shield of California, Oakland, California, USA.; ^3^ West Health Institute, La Jolla, California, USA.

**Keywords:** home-based palliative care, palliative care, utilization

## Abstract

**Background:**

A California-based health plan offered home-based palliative care (HBPC) to members who needed support at home but did not yet qualify for hospice.

**Objectives:**

This study compares hospital and emergency department (ED) utilization and costs and mortality for individuals receiving HBPC to a cohort not receiving palliative care services (Usual Care).

**Design:**

This is an observational retrospective study using claims data covering a prestudy period and a study period during which time half of the study population received HBPC services.

**Setting/Subjects:**

Seriously ill individuals who received HBPC were matched with those receiving Usual Care using a propensity-based matching algorithm.

**Intervention:**

Interdisciplinary teams from home health and hospice agencies provided HBPC services.

**Measurements:**

Outcome measures included hospital and ED utilization and cost before and during the study period and mortality during the study period.

**Results:**

For both groups, hospital and ED utilization and associated costs were higher during the prestudy period than during the study period. No differences were found in outcome measures between groups during the study period. Average time in the study period was longer for the HBPC group than that in the Usual Care group, indicating that they lived longer or transitioned to hospice later.

**Conclusion:**

Although individuals in both groups were living with serious illnesses for which worsening health and increased acute care utilization are expected over time, both groups had reduced acute care utilization and costs during the study period compared with the prestudy period. Reduced utilization and costs were equivalent for both groups.

## Introduction

Decades of research shows that palliative care can be effective in reducing pain, controlling symptoms, and improving quality of life, while also decreasing health care utilization and the costs of medical care.^1–5^ Palliative care interventions can also reduce the number of ICU admissions and reduce ICU length of stay.^6^ Patients who receive palliative care in their homes tend to have lower hospital utilization rates and lower costs of care during the past three to six months of life^7–9^ as well as lower emergency department (ED) usage and higher satisfaction with care.^7^

Findings from Cassel et al. demonstrated that hospital costs and total costs per month were lower for patients enrolled in a home-based palliative care (HBPC) program, as compared with propensity-matched control patients with the same chronic conditions. In the final months of life, only slight increases in costs were seen for patients in the HBPC program, compared with dramatic increases in costs for those in the comparison group.^8^ The randomized controlled trial from Brumley et al. showed that patients randomized to HBPC had higher rates of satisfaction, reduced ED and hospital utilization, and lower costs than controls receiving usual palliative care.^7^

Given the benefits associated with in-home palliative care, one large insurance plan in California aimed to increase its capacity to deliver in-home palliative care services to members with serious illnesses whose referring provider “would not be surprised” if the member died in the next year and who was not eligible for or interested in hospice services. The insurer contracted with a network of providers who were able to deliver palliative care in the home and providers were reimbursed through a monthly case rate for each enrolled member.

Contracted providers were required to have an interdisciplinary palliative care team, including a physician, nurse practitioner, nurse, social worker, and chaplain. This team provided pain and symptom management, disease management, education, advance care planning, psychosocial support, grief counseling, spiritual counseling, and coordination of care including assistance getting needed medication, supplies, and durable medical equipment (DME). HBPC providers were not required to be able to do home visits 24/7 but they were required to have a 24/7 phone line. The frequency of visits provided by team members varied depending on patients' needs, but a physician or nurse practitioner was required to see each patient at least every three months and any time there was a significant change in the goals of care.

The objective of this study is to assess whether acute health care utilization and costs decreased after members were enrolled in the health plan's HBPC program.

## Methods

### Inclusion and exclusion

To assess the impact of participation in HBPC on costs and utilization, a retrospective analysis of three years of health plan claims data was conducted (2017–2019). The data set contained utilization information for 2069 members enrolled in HBPC (HBPC group) and identified a matched control group from a database of 50,000 members who were not enrolled in HBPC (Usual Care group). Members included in the analysis all had a serious illness diagnosis of heart failure, lung disease, cirrhosis, dementia, renal failure, HIV, cancer, neurodegenerative disease, stroke, advanced age, and/or frailty.

To be included, members also had to have one qualifying event defined as one or more ED visits or inpatient hospital stays within the past 12 months and had to be living in the community and not enrolled in hospice. Members were also required to have 12 months of consecutive enrollment with the health plan before enrollment in the HBPC program or the pseudo-enrollment date for the Usual Care group.

Members enrolled in a different health plan program that provided home-based services to individuals with five or more chronic medical conditions were excluded from both the HBPC group and the Usual Care group. All members were either enrolled in a preferred provider organization (PPO), administrative services only (ASO), health maintenance organization (HMO), or Medicare Advantage (MA) health plan.

### Cohort construction

**FIG. 1.**
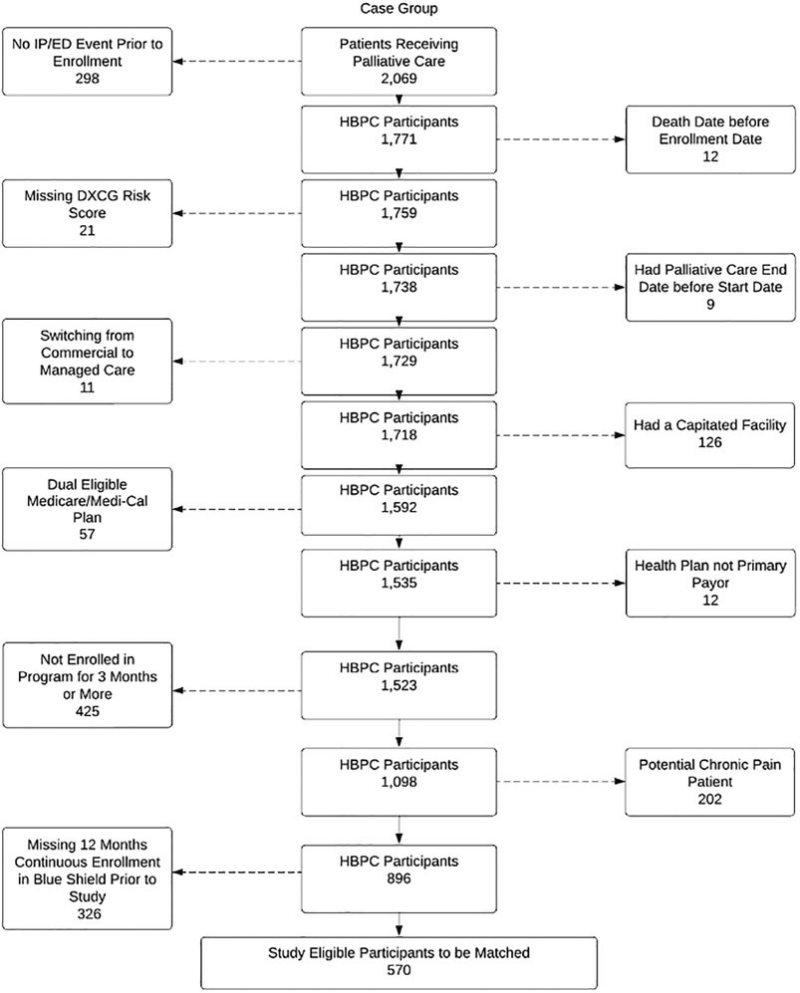
HBPC group inclusion and exclusion criteria. HBPC, home-based palliative care.

Two cohorts were created. One with members enrolled in HBPC (HBPC group) and another with those who met inclusion criteria but were not enrolled in HBPC (Usual Care group). Figure 1 shows how inclusion and exclusion criteria were used to create the HBPC group (570 members) and the Usual Care group (24,311 members).

### Enrollment and pseudo-enrollment

**FIG. 2.**
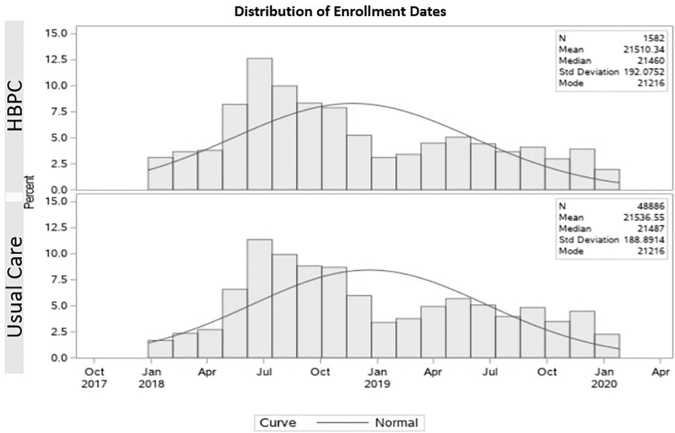
Distribution of enrollment dates for HBPC group and pseudo-enrollment dates for usual care. HBPC, home-based palliative care.

In the HBPC group, the hospital and ED costs and utilization in the 12 months preceding program enrollment (prestudy period) were compared with the period during which the patient received HBPC (study period). The study period started the day after enrollment in the HBPC program. Because the Usual Care group did not have an enrollment date (since they were never enrolled in a HBPC program), a pseudo-enrollment date was created for each person in the Usual Care group using the following method: the distribution of enrollment dates from the HBPC group was randomly applied to the Usual Care group and used to set pseudo-enrollment dates to create a Usual Care group whose distribution of enrollment dates was similar to the enrollment dates of the HBPC group (see Fig. 2).

Finally, pseudo-enrollment dates for the Usual Care group were constrained so that they occurred after a qualifying event (ED visit or hospitalization) to match the criteria used to enroll participants in the HBPC group.

### Defining propensity score matching algorithm

The 12-month period before the enrollment date was used to identify a propensity-matched Usual Care group patient for each patient in the HBPC group. A logistic regression model was used to calculate a propensity score for inclusion in the Usual Care group. The model's covariates included the Diagnostic Cost Group (DxCG)^10^ that is a proprietary score used by the health plan to predict resource consumption and cost using age, gender, chronic and acute conditions, and the Charlson Comorbidity Index scores,^11^ which is a weighted index used to predict risk of death within one year of hospitalization for patients with comorbid conditions.

Other covariates used were age, gender, total number of inpatient claims, total ED claims, total cost, durable medical equipment cost, dialysis cost, and line of business (PPO, ASO, HMO, MA plan). The greedy nearest neighbor one-to-one matching algorithm using the PSMATCH procedure in SAS Studio Version 3.71 (SAS Institute, Inc.) was used. This algorithm selects the individual in the Usual Care group that is nearest to an individual in the HBPC group on a one-to-one basis without replacement.

Exact matches were required for DME use, dialysis, and health plan type since these subsets of members were thought to be different from the groups at large (e.g., DME was used as a proxy for functional status; dialysis is expensive and has a significant impact on cost; payment varied between diagnostic-related groups (DRG) and per diem depending on the health plan type). After matching on the factors above, the algorithm was applied, and one HBPC group member was matched to the one Usual Care group member who most closely matched in disease profile and utilization pattern during the prestudy period.

### Study period

The study period started on the (pseudo) enrollment date and ended when patients either (1) entered hospice, (2) died, or (3) if neither of these occurred, then the final date of the study period, which was December 30, 2019. Although the prestudy period was a standardized 12 months, the length of the study period varied in both groups since there were a variety of ways the study period could end. The variability in enrollment time was normalized by reporting all utilization and costs as per member month per 1000 members.

### Outcome variables, study design, and analyses

The outcome variables of interest for this study were (1) per member per month cost of acute care (ED and hospital costs), (2) ED utilization (ED claims per member per month per 1000 patients), (3) hospital utilization (inpatient claims per member per month per 1000 patients), and (4) mortality. Cost data were limited to inpatient hospital and ED costs because those were the only reliable cost sources across all health plan types (PPO, ASO, HMO, and MA). As such, the “cost of acute care” variable is the sum of ED and hospital inpatient costs only.

Two sets of analyses were completed. First, outcome variables were compared between the HBPC group and the Usual Care group during the study period. Second, the difference in these outcome variables was assessed between the prestudy and study periods within the HBPC and Usual Care groups.

### Statistical testing

All statistical tests were conducted with SAS Studio Version 3.71 (SAS Institute, Inc.). To compare the differences between (HBPC vs. Usual Care) and within (pre/post) each group, we utilized the Mann–Whitney *U* nonparametric test. This nonparametric test was used to account for the nonstandard distributions of all outcome variables. All tests were performed using the *p* < 0.05 indicated significance level.

## Results

### Descriptive statistics

A summary of the descriptive statistics by group (Table 1) shows that before propensity score matching, observations in the groups varied in several ways, including demographics, comorbidities, DxCG risk scores, utilization, cost, and lines of business. Propensity score matching created 570 matched pairs, and more evenly balanced groups as evidenced by the standardized differences of <0.1 for all covariates across groups.

**Table 1.** Covariate Balance Across Home-Based Palliative Care and Usual Care Groups Before and After Propensity Score Matching

	Pre-study period					
	Pre-matched sample			Propensity-score matched sample		
Variable	Mean HBPC group (*n = *571)	Mean Usual Care group (*n = *24,311)	Standardized difference	Mean HBPC group (*n = *570)	Mean Usual Care group (*n = *570)	Standardized difference
Age	66.4	52.9	0.90	66.4	67.0	−0.04
DxCG score	11.3	5.8	0.57	11.3	10.4	0.08
Charlson Comorbidity Index	5.6	3.2	0.76	5.4	5.5	−0.03
Inpatient visits	1.3	0.4	0.63	1.3	1.1	0.08
Admission days	7.5	1.9	0.39	7.6	6.6	0.06
ED visits	1.0	0.9	0.12	1.0	0.9	0.10
Total cost of care	$78,155	$30,646	0.41	$78,240	$74,389	0.02
% HMO	17.2	26.6	−0.23	17.2	17.2	0.00
% ASO/PPO	39.2	66.8	−0.58	39.3	39.3	0.00
% Medicare advantage	43.6	6.6	0.94	43.5	43.5	0.00
% Female	53.2	59.0	−0.12	53.2	51.9	0.02

ASO, administrative services only; DxCG, Diagnostic Cost Group; ED, emergency department; HBPC, home-based palliative care; HMO, health maintenance organization; PPO, preferred provider organization.

### HBPC group prestudy period versus study period

Analyses revealed fewer ED visits for members enrolled in the HBPC program during the study period (851 per member months per 1000 members) as compared with during the prestudy period (1034 per member months per 1000 members), fewer hospitalizations during the study period (1069 per member months per 1000 members) as compared with the prestudy period (1255 per 1000 members), and lower cost of care in the study period ($2746 per member per month) as compared with the prestudy period ($3996 per member per month). See Table 2.

**Table 2.** Home-Based Palliative Care Group Prestudy Period Versus Study Period Outcomes

	Prestudy period (*N = *565)				Study period (*N = *565)				
	Mean	Median	Min	Max	Mean	Median	Min	Max	*p*
Inpatient visits (per member months per 1000 members)	1255	1000	0	14,000	1069	0	0	19,864	<0.0001
ED visits (per member months per 1000 members)	1034	1000	0	14,000	851	0	0	16,174	<0.0001
ED and hospital costs PMPM	$3996	$1204	$0	$114,885	$2746	$45	$0	$182,220	0.0001

PMPM, per member, per month.

### Usual Care prestudy period versus study period

Analyses revealed fewer ED visits for members in the Usual Care group during the study period (728 per member months per 1000 members) as compared with the prestudy period (1894 per member months per 1000 members), fewer hospitalizations during the study period (947 per member months per 1000 members) as compared with the prestudy period (1136 per member months per 1000 members), and lower cost of care in the study period ($2918 per member per month) as compared with the prestudy period ($3448 per member per month). See Table 3.

**Table 3.** Usual Care Group Prestudy Period Versus Study Period Outcomes

	Prestudy period (*N = *565)				Study period (*N = *565)				
	Mean	Median	Min	Max	Mean	Median	Min	Max	*p*
Inpatient visits (per member months per 1000 members)	1136	1000	0	22,000	947	0	0	62,000	<0.0001
ED visits (per member months per 1000 members)	894	1000	0	13,000	728	0	0	14,099	<0.0001
ED and hospital costs PMPM	$3448	$845	$0	$188,425	$2918	$0	$0	$194,551	<0.0001

### HPBC versus Usual Care between-group comparison results

Though the difference in mortality between the two groups came close to reaching statistical significance with a trend toward a longer length of life in the HBPC group, the difference was not statistically significant. The analyses revealed no significant differences in acute care utilization or cost of acute care variables between the HBPC and Usual Care groups (Table 4). It is notable that there was a statistically significant difference in total months enrolled during the study period. The HBPC members had a slightly longer study period, indicating that they either lived longer or transitioned to hospice after a stay in the HBPC program that was longer than the stay of members in the Usual Care group.

**Table 4.** Home-Based Palliative Care Versus Usual Care Between-Group Outcomes

	HBPC group		Usual Care group		Wilcoxon two-sample test
	Mean	Median	Mean	Median	*p*
Mortality	10%	0%	14%	0%	0.066
Total months enrolled	11.9	12.4	11.0	10.3	0.01
Inpatient visits (per 1000 patients)	947.1	0.0	1069	0.0	0.26
ED visits (per 1000 patients)	728.1	0.0	851.4	0.0	0.15
ED and inpatient costs PMPM	$2846	$0	$2741	$43	0.15

### Limitations

The use of claims data to match patients has limitations. Though claims data can be used to match members based on some factors, it cannot be used to match members on other factors that may be of particular importance including functional status and social barriers to health care. These two factors are primary indicators in the clinical selection of patients who will most benefit from HBPC. It is possible that there are differences in these factors across the two study groups. Including these factors in a prospective study might lead to different results.

Furthermore, utilization and costs were not measured during the last six months of life when it is well documented that costs increase. Previous research has indicated that palliative care can generate cost savings during this period.^7–9^ Unfortunately, the last six months of life was a period that was under-represented in this study preventing a meaningful analysis of health care cost and utilization during the time when there may be the most opportunity for savings. The last six months of life can only be identified after an individual dies and the number of deaths during the study period was too low to perform a meaningful analysis on this subgroup.

If the last six months of life had been captured for more study participants, a greater impact of the program may have been demonstrated. The fact that so few members died during the study period suggests that the members were receiving upstream palliative care.

In addition, it is possible that the health plan members with the greatest potential to show decreased utilization and cost were excluded from the study because the health plan had another home-based program for high-cost members with five or more chronic conditions, and these members were excluded from this study.

The fact that the HBPC intervention was delivered by multiple providers may have introduced variability in the way in which the intervention was implemented. Having one provider implement the intervention would lead to greater consistency.

The impact of the payment methodology used to reimburse the HBPC providers should also be considered. It is possible that using a case rate instead of a fee-for-service model incentivized fewer interventions and less frequent visits on the part of the HBPC provider. A home-based intervention that incentivized more contact with the member might have been needed to show improvement in utilization and cost during the study period.

Because rates and payment methodology vary based on the plan type (PPO, ASO, HMO, and MA), costs of acute care may also vary. This study did not include a subgroup analysis based on the plan type because the sample size was not large enough to show statistical significance results in a subgroup analysis.

Finally, palliative care can lead to improved quality of care including better pain and symptom management and quality of life.^12–14^ This study lacks data on these factors. It is important to understand the degree to which pain and symptom management and quality of life were impacted by enrollment in HBPC. If these factors were improved as compared with the Usual Care group, one might conclude that offering HBPC is important and beneficial to patients regardless of whether it leads to cost savings.

## Discussion

Few past studies of HBPC include a control group, and/or account for the natural variation in health care utilization that occurs over time (regression to the mean). Enrollment in palliative care programs often occurs due to high health care utilization and/or an expensive encounter such as a hospitalization. As such, at the time of enrollment, health care utilization and the cost of care are often at an extreme. Regression to the mean predicts that for many patients, the natural tendency will be for those variables to return to less extreme highs regardless of any intervention. One must be careful, then, not to confuse normal regression to the mean with the actual impact of a particular intervention.

A strength of this study was the use of a propensity-matched comparison group (Usual Care group) to account for regression to the mean. Because there were no differences between outcome variables between the HBPC and Usual Care groups, it is likely that the utilization and cost differences between the prestudy period and the study period for the HBPC group were not specifically related to the services received as part of the HBPC program, but rather due to natural variation in utilization and associated costs.

The fact that costs of care went down for both the HBPC group and the Usual Care group during the study period is notable as patients with serious illnesses typically decline and have more disease burden over time, increasing utilization and cost of care. The result suggests that members in this study were receiving the HBPC intervention before the last months of their lives when health care costs are known to rise. There was a trend toward lower utilization and cost in the HBPC group, and if the study period had been longer, then reduction in utilization and cost may have been seen as the members' underlying medical conditions worsened.

It is also notable that patients receiving the HBPC intervention had a longer study period, suggesting that they either lived longer or spent longer on the HBPC before transitioning to hospice. This, combined with the fact that the lower mortality rate in the HBPC group was approaching statistical significance, suggests that further studies should look at the impact of HBPC on mortality.

## Conclusions

This study used claims data to assess utilization and the cost of acute care for a group of patients who received HBPC. A within-group analysis showed that both utilization and the cost of care were higher in the prestudy period than in the period during which patients received HBPC. However, a propensity-matched control sample that did not receive HBPC also had reduced utilization and cost of care between the prestudy and study periods. Though the decrease in cost of care was greater for the HBPC group than for the standard care group, it was not statistically significant.

A prospective control study with a single HBPC provider that accounts for functional status and social determinants of health as well as the other factors controlled for in this study and that follows patients through the end of their life is needed to better understand the impact of HBPC on utilization, cost of care, and mortality. Palliative care is known to improve important quality-of-life factors including pain and symptom management. As such, being able to deliver HBPC to patients with serious illness without significant impact on costs of care may be sufficient to suggest that doing so creates value in the health care system.

## References

[1] Casarett D, Pickard A, Amos BF, et al. Do palliative consultations improve patient outcomes? J Am Geriatr Soc 2008;56(4):593–599; doi: 10.1111/j.1532-5415.2007.01610.x 18205757

[2] Finlay IG, Higginson IJ, Goodwin DM, et al. Palliative care in hospital, hospice, at home: Results from a systematic review. Ann Oncol 2002;13:257–264; doi: 10.1093/annonc/mdf668 12401699

[3] Gade G, Venohr I, Conner D, et al. Impact of an inpatient palliative care team: A randomized controlled trial. J Palliat Med 2008;11(2):180–190; doi: 10.1089/jpm.2007.00554 18333732

[4] Higginson IJ, Finlay I, Goodwin DM, et al. Do hospital-based palliative teams improve care for patients or families at the end of life? J Pain Symptom Manage 2002;23(2):96–106; doi: 10.1016/S0885-3924(01)00406-7 11844629

[5] Higginson IJ, Finlay I, Goodwin DM, et al. Is there evidence that palliative care teams alter end-of-life experiences of patients and their families? J Pain Symptom Manage 2003;25(2):150–168; doi: 10.1016/S0885-3924(02)00599-7 12590031

[6] Khandelwal N, Kross EK, Engelberg RA, et al. Estimating the effect of palliative care interventions and advanced care planning on ICU utilization: A systematic review. Crtic Care Med 2015;43(5):1102–1111; doi: 10.1097/CCM.0000000000000852 PMC4499326 25574794

[7] Brumley R, Enguidanos S, Jamison P, et al. Increased satisfaction with care and lower costs: Results of a randomized trial of in-home palliative care. J Am Geriatr Soc 2007;55(7):993–1000; doi: 10.1111/j.1532-5415.2007.01234.x 17608870

[8] Cassel BJ, Kerr KM, McClish DK, et al. Effect of a home-based palliative care program on healthcare use and costs. J Am Geriatr Soc 2016;64(11):2288–2295; doi: 10.1111/jgs.14354 27590922 PMC5118096

[9] Lustbader D, Mudra M, Romano C, et al. The impact of a home-based palliative care program in an accountable care organization. J Palliat Med 2017;20(1):23–28; doi: 10.1089/jpm.2016.0265 27574868 PMC5178024

[10] Ash A, Porell F, Gruenberg L, et al. Adjusting Medicare capitation payments using prior hospitalization data. Health Care Financ Rev 1989;10(4):17–29. 10313277 PMC4192932

[11] Charlson M, Pompei P, Ales KL, et al. A new method of classifying prognostic comorbidity in longitudinal studies: Development and validation. J Chronic Dis 1987;40(5):373–383. 3558716 10.1016/0021-9681(87)90171-8

[12] Kittelson SM, Elie MC, Pennypacker L. Palliative care symptom management. Crit Care Nurs Clin North Am 2015;27(3):315–339; doi: 10.1016/j.cnc.2015.05.010 26333754

[13] Meier DE, Brawley OW. Palliative care and the quality of life. J Clin Oncol 2011;29(20):2750. 21670456 10.1200/JCO.2011.35.9729 PMC3139393

[14] Wilkie DJ, Ezenwa MO. Pain and symptom management in palliative care and at end of life. Nurs Outlook 2012;60(6):357–364. 22985972 10.1016/j.outlook.2012.08.002 PMC3505611

